# Flow Cytometric Methods for Indirect Analysis and Quantification of Gametogenesis in *Chlamydomonas reinhardtii* (Chlorophyceae)

**DOI:** 10.1371/journal.pone.0161453

**Published:** 2016-09-27

**Authors:** Catherine E. Seed, Joseph L. Tomkins

**Affiliations:** Centre for Evolutionary Biology, School of Animal Biology (M092), The University of Western Australia, Crawley, Australia; Wuhan University, CHINA

## Abstract

Induction of sexual reproduction in the facultatively sexual *Chlamydomonas reinhardtii* is cued by depletion of nitrogen. We explore the capacity for indirect monitoring of population variation in the gametogenic process using flow cytometry. We describe a high-throughput method capable of identifying fluorescence, ploidy and scatter profiles that track vegetative cells entering and undergoing gametogenesis. We demonstrate for the first time, that very early and late growth phases reduce the capacity to distinguish putative gametes from vegetative cells based on scatter and fluorescence profiles, and that early/mid-logarithmic cultures show the optimal distinction between vegetative cells and gamete scatter profiles. We argue that early/mid logarithmic cultures are valuable in such high throughput comparative approaches when investigating optimisation or quantification of gametogenesis based on scatter and fluorescence profiles. This approach provides new insights into the impact of culture conditions on gametogenesis, while documenting novel scatter and fluorescence profile shifts which typify the process. This method has potential applications to; enabling quick high-throughput monitoring, uses in increasing efficiency in the quantification of gametogenesis, as a method of comparing the switch between vegetative and gametic states across treatments, and as criteria for enrichment of gametic phenotypes in cell sorting assays.

## Introduction

*Chlamydomonas reinhardtii* is a unicellular, isogamous alga of the Chlorophyceae, which displays facultative sexual reproduction; able to switch between asexual and sexual modes of reproduction. *Chlamydomonas* is a widely used model in areas such as biofuel production [1, 2] flagella biology [3, 4], and as a model for the evolution and maintenance of sexual reproduction [5, 6]. Naturally occurring in soil and fresh water, the facultative nature of *Chlamydomonas* reproduction is understood to be an adaptation to harsh environments. Granick Sager and Granick (7) initially demonstrated that gametogenesis in this species was cued by depletion of nitrogen in the environment, with high nitrogen availability inhibiting gametogenesis.

Reproducing via mitotic asexual division in environments of nutrient abundance, cells possess one of two non-recombining mating-type regions, leading to the requirement for fusion of cells of opposite mating types in sexual reproduction upon a Nitrogen level decrease [8]. During gametogenesis, *Chlamydomonas* undergoes a mitotic division, producing gametes capable of fusing to produce recombinant offspring, each displaying flagellar proteins termed agglutinins, integral to gamete fusion [8, 9]. Once fusion of opposite mating types has occurred—resulting in the production of a diploid, zygote—meiosis occurs, leading to the production of recombinant offspring [10–13].

Gametogenesis can broadly be divided into two processes; the conversion to pregametes [14], and the acquisition of mating competence [15–17]. A detailed picture of the transcriptional programs involved in gametogenesis, including the associated transcriptional changes associated with mating competence is emerging [18–20]. Previous analyses have demonstrated variability of mating efficiency within lines and between clones [21], however, the exact nature of this variation, and its relationship to gametogenesis and cell morphology remain unclear. Therefore, high-throughput and multi-parameter methods are required for a robust quantification of mating efficiency, efficient and repeatable production of gametes, competitive mating methods, as well as a screening method for gametic and sexual mutants, and cell sorting to obtain clonal and axenic populations derived from gametic cells [22].

*Chlamydomonas reinhardtii* divides asexually every 4–8 hours under conditions of continuous light. In early/mid G1 phase, cells pass a size checkpoint controlling transition through the cell division cycle; a minimum size must be attained for asexual division, estimated at 2.2 times the post mitotic cell size [23], which can be moderated by light regime [24]. Craigie and Cavalier-Smith (24) showed that in mitotic divisions, daughter cell size is uniform and independent of parent cell volume, suggesting a minimum volume of daughter cells. By adopting alternating light cycles, cell cycles can be synchronised [25], enabling successive rounds of division based on cell size to occur only in the dark cycle. This capacity for synchronisation makes *Chlamydomonas* an attractive model for exploring individual differences in the capacity to undergo gametogenesis; traits which have previously been less amenable to analysis due to the low resolution methods available. The capacity for a cell to undergo gametogenesis under synchrony (where cell division is limited to a dark phase), is understood to be determined both by a cells capacity to undergo cell division after environmental nitrogen levels decrease [26], in addition to a cells ability to detect the decreasing nitrogen levels in the environment; the second of which there has been relatively little investigation.

It is expected that the phase of growth of a cell under study may affect the scatter and fluorescence changes detected under experimental conditions. From exponential to stationary stages, changes in cell morphology such as decreases in cell size, physiology and gene expression are expected to occur. A previous study in *E*. *coli* identified 20 cell types representing different stages of differentiation and growth [27], while other studies have shown growth phase dependent enzymatic production [28]. Earlier investigations in *Chlamydomonas* show growth phase related changes in intracellular components such as carbohydrate:protein and lipid;protein ratios [29]. Growth phase dependent changes occur in physiology and behaviour, such as autophagy which is upregulated in stationary phase and down regulated upon induction of log phase [30], and phototaxis which is highest under conditions of exponential growth [31]. The deprivation of nutrients, such as phosphate, sulphur, magnesium, CO_2_, potassium and the gametogenic cue nitrogen, (which is expected to increase stress under later growth phases) has been shown to lead to changes including glycogen accumulation [32, 33]. It is expected that under nutrient stress, cells will prioritise the accumulation of storage products. As a result it is important to investigate whether such processes make vegetative cells morphologically more similar to gametes or whether such environmental changes can lead to cells undergoing gametogenesis (due to decreases in nitrogen levels).

While there has been a great deal of interest in the process of gametogenesis, it remains difficult to identify and therefore quantify gametes within a population. Given the environmental cue required for gametogenesis, we would expect population variation in the perception of the cue and initiation of gametogenesis, and populations may therefore be a mixture of vegetative cells, cells undergoing gametogenesis, and gametes. Attempts to create gamete mating-type-specific agglutinin antibodies specific enough to repeatedly identify and enrich for gametes has been difficult, with many of the antigens present in gametic agglutinins showing domain crossover with other cell wall proteins found in gametes and non-gametic cells [34], limiting the applicability of immuno-methods for enriching gametes cells. Previous approaches to studying gametogenesis have relied on microscopic analyses and mating efficiency tests to determine when mating competent cells emerge within a population [35]. This approach is time-consuming and requires extrapolation from small samples to larger populations. We sought to explore whether scatter and fluorescence profiles of cells measured using flow cytometry, show repeatable changes during the process of gametogenesis, and whether this may offer an alternative method for exploring gametogenesis, providing single cell quantitative resolution of cellular properties over the process of gametogenesis with large sample sizes. Given that flow cytometry enables addition of fluorescent stains and immuno-methods, this method could enhance our understanding of the process of gametogenesis. Secondly, by identifying parameters to distinguish individual vegetative and gamete cells in a population, it may be possible to quantify the relative number of cells of each type, and to explore the dynamics and genetics underlying the decision to become a gamete.

Flow cytometry is a mechanised technique involving a hydrodynamic focussed fluid stream of single cells which is passed through a series of laser beams, collecting fluorescence and light scattering properties as the cells scatter light while passing through the light stream [36]. Forward-scatter is a measure of light scatter detected by a photodiode in line with the excitation light, and often provides a relative indication of cell size [37]. Light which is scattered largely from its original path is collected by a second detector situated orthogonally to the emitting beam [37]. Dichroic mirrors are used to split this light. Scattered excitation light collected through the orthogonally situated detectors is termed side-scatter and provides an indication of internal and external structure/complexity in addition to the refractive index of the cell [37]. However, the precise relationship between scatter and cell characteristics may differ depending on the cell type. Optical filters are used to detect specific ranges of scattered light which can be quantified, before the scattered light and emitted fluorescence are detected by the photomultiplier tubes [37]. Collection of such fluorescence intensities allows the detection of experimentally introduced dyes or labels, which are excited by a low wavelength laser, and which fluoresce at higher wavelengths [36, 38, 39]. However, these studies can be affected by naturally fluorescent pigments in cells under analysis. This auto fluorescence is often seen as an issue for flow cytometric analyses, with auto fluorescent signals overlapping into fluorophore channels which must be compensated for.

Complex changes occur throughout the process of *Chlamydomonas* gametogenesis in terms of size, subcellular structure and emission spectra of auto fluorescent pigments. More widely in biology, different isotypes or specific combinations of pigments seen in different species, or even between cell types of the same species have been proposed as possible signatures with which we can identify specific cell populations [40], and the auto fluorescent properties of *Chlamydomonas* gametes indicate a candidate for such an approach. Due to naturally fluorescent compounds such as NADPH, chlorophyll a and b (which are excited by the 488 laser), flow cytometric monitoring of scattering properties and fluorescence has been shown to be able to distinguish algal species [39, 41, 42]. While endogenous compounds may limit the fluorescent dyes and labels compatible with algal studies [39], their auto fluorescent properties, rather than being a barrier to analyses, may in fact act as a substitute for externally applied fluorophores.

Here we show how auto fluorescence in *Chlamydomonas* (which fluoresces highly in the red, far red and infra-red spectra [39] and lower in other channels), may allow monitoring of differentiation and the distinguishing of different differentiated cell types within the same population. We demonstrate the utility of flow cytometry for high throughput quantification of gametogenesis, and highlight the potential of this technique to allow for detailed approaches to studies of gametogenesis and the decision to become a gamete. We use these techniques to explore optimal conditions for studying gametogenesis. We show how flow cytometry relates to earlier findings applying mating capacity and manual mating counts methods to studies of gametogenesis in *Chlamydomonas*, and therefore whether flow cytometry can serve as a proxy for understanding efficiency and variation in gametogenesis. Finally we explore the relationship between gamete and vegetative characteristics across the growth curve of *Chlamydomonas* cultures.

## Materials and Methods

### Strains and culture conditions

Strain CC-125 of *Chlamydomonas reinhardtii* for use in gametogenesis time courses was obtained from the *Chlamydomonas* Resource Centre (University of Minnesota). Additional strains CC-1690, CC-1692, CC-124, CC-2935, CC-2936, CC-2931 and CC-2932 (for CEROS computer-assisted sperm analysis system (*CASA* and comparative analyses) were also obtained from the CRC. Cells were maintained for long term storage on Tris-Acetate-Phosphate (TAP) [43] agar plates supplemented with yeast extract. TAP media was inoculated with cells taken directly from agar plates. All liquid and agar cultures were maintained at 22±1°C [44] with a 12:12 light/dark diurnal photoregime under ‘cool white’ fluorescent bulbs [8]. Liquid cultures were agitated using an orbital shaker at 120rpm. All cell concentration measures were calculated using a Haemocytometer counting chamber using the fixative Lugol’s solution as described in [8].

### Vegetative and gametic comparisons

For vegetative and gametic fluorescence comparisons, three biological replicates of line CC-125 were grown in pre-growth cultures for three days, re-diluted to 500,000 cells/ml and grown for a further three days to ensure similarity in phase of growth; in the early logarithmic phase. Line CC125 was chosen as it shows capacity to mate with CC-124 and is amenable to mating trials (unpublished data). All cell concentration measures were calculated using a haemocytometer counting chamber using the fixative Lugol’s solution as described [8]. 500ul samples of 1) vegetative growths, 2) cells immediately after centrifugation (centrifuged at 3000g for 2 min and resuspended in double distilled water (ddH_2_0), and 3) cells 24 hours after resuspension in (ddH_2_0), were each fixed in 500ul 2% paraformaldehyde (PFA), to create 1% final concentration of fixative. A second experiment repeated these methods, with the adjustment that condition 2 used cells immediately after centrifugation and resuspended in TAP media. This analysis confirmed no significant effect of centrifugation on scatter or fluorescence profiles (S1 Fig). Thus we can assume that the cells remain intact after centrifugation and, that changes in scatter and fluorescence after 24hours of nitrogen removal can be attributed to gametogenesis.

### Gametogenesis time-courses

In the gametogenesis time-course analyses, two biological replicates of line CC-125 were grown directly from plates for seven days for late logarithmic cultures (~4–5 x 10^6^ cells /ml). This enabled subsequent testing of effect of gametogenesis by applying t tests using two independent cultures that each had ~20,000 independent flow cytometric data points. The ~20,000 data points were combined to give a mean value at the beginning and end of the time-course for each independent culture. Late logarithmic cultures were chosen for the detailed time course due to historical use of this phase in mating experiments of *Chlamydomonas* species [11, 45–48]. To investigate the effect on growth phase on the phenotype of gametes, two biological replicates were grown for three days for the early logarithmic cultures (~1 x 10^6^ cells /ml). Haemocytometer counts were taken before experiments for vegetative growth phase verification, and cells were dilute to 500,000cells/ml to avoid high concentrations in flow analysis.

To investigate the effect of phase of light on gametogenesis, at the initiation of the light cycle, cells from two late logarithmic replicates were aliquoted, centrifuged at 3000g for 2min and resuspended in ddH_2_0 which is nitrogen free. Full randomisation was employed. The same process was repeated mid-way through the 12 hour light cycle for all remaining samples to induce gametogenesis.

For vegetative and gametic comparisons, 500ul samples of three time-points; vegetative, 0hr (immediately after transfer to ddH_2_0) and 24hr post ddH_2_0 transfer, were fixed in 500ul 2% PFA and maintained at 4°C. ddH_2_0 was used to eliminate any effect of trace nitrogen present in the trace elements solution and TRIS used to create low nitrogen media, which may affect the induction of gametogenesis. Samples were then collated back into the conditions and randomised. Samples were taken hourly over 24hours for the late logarithmic gametogenesis time course. Samples were taken every six hours for two replicates each of early and late logarithmic phase samples and two replicates of samples testing the effect of time in light cycle nitrogen removal was performed. Cultures were maintained under continuous illumination over the course of gametogenesis.

250ul Samples were taken and fixed in 250ul 2% PFA. These were stored at 4°C until analysis. Prior to all analyses samples were filtered using 40um filters. In all analyses, time 0hr samples were taken immediately after transfer to ddH_2_0 to limit scatter effects of media on flow cytometric assessment and the effect of centrifugation on forward-scatter values.

To investigate the effect of growth phase on gametogenesis, three replicates samples of similar inoculum size from line CC-125 *Chlamydomonas* were inoculated into separate test tubes of 10ml TAP directly from a single 1.5% agar plate. Samples were then randomised. Each day for 8 days, 100ul samples were taken from each vegetative growth and fixed in Lugol’s solution for quantification using a haemocytometer to determine vegetative phase of growth. 500ul Samples were also taken from each tube of live cells, centrifuged at 3000g for 1min and resuspended in 600ul ddH20. 100ul of this solution was fixed in Lugol’s solution for quantification of post transfer concentration. The nitrogen free suspension was kept in continuous light for 24hours, after which 100ul was fixed in Lugol’s solution for quantification of the gametic population. The remaining 400ul was fixed in 400ul of 2% PFA for flow cytometric analysis and stored at 4C. Concentrations of the vegetative, post nitrogen transfer and post gametic samples were measured using a haemocytometer. Samples were filtered through a 40um filter before flow cytometric analysis

### CASA

Relative cell size analyses were obtained using three replicates of ten cell lines (CC-1690, CC-1692, CC-125, CC-124, CC-2935, CC-2936, CC-2931, CC-2932, CC-2343, CC-2342). Replicate populations were derived from the sample plate of the respective cell line, and grown in separate pre-growth 10ml tube environments for three days under described growth conditions. Cells were then resuspended at 250,000 cells/ml for a further three days growth. Relative cell size was quantified using the CEROS computer-assisted sperm analysis system (*CASA*) (v.10, *Hamilton & Thorne Research*) using parameters optimised for *Chlamydomonas*. Individual database text files with track details were generated for every cell analysed. At early logarithmic phase, two 250ul samples were taken from each population. One was measured immediately using *CASA* to indicate relative vegetative size and cell concentration. The second was centrifuged at 3000g for 2min and resuspended in ddH_2_0 for 24hours, when measurements were again taken to indicate relative gametic size and cell concentration using a haemocytometer.

### Flow cytometry

All flow cytometric analyses were conducted on a FACS *Calibur* flow cytometer (*BD Biosciences*) with an air-cooled argon laser (488nm emission), a red emitting diode (635nm emission) and four filters and detectors. For all morphological and auto fluorescent analyses, 20,000 cells were sampled per condition. In the gametogenesis time-course samples, where sample concentration limited data collection, samples were only included if they exceeded 15,000 events in the ‘Cell’ population. For estimation of proportion of dead cells, live samples were filtered immediately before analysis, when 2ug/ml Propidium Iodide (PI) was added in dark for 10min before measurement and percentage dead cells was calculated (average = 2.45% dead cells) (S2 Fig). All flow cytometric samples were filtered through 40um filters before analysis. Bleach was run between each sample until contaminating cells were not detected. PI stock solutions were filtered through 0.22um filters to remove small particles and stored at -20°C before use.

#### DNA ploidy analysis

Optimisation of the ploidy protocol was determined using mid-logarithmic synchronised vegetative cells prior to, 1 hour after and 2 hours into the dark phase of the 12:12 light: dark cycle as under synchronisation, cells only divide in the dark phase (S3A Fig).

Concentrations and combinations of stains, fixatives and permeabilisation agents were optimised. For ploidy determination during gametogenesis, two 30ml replicates were grown for three days to produce two early logarithmic cultures (1–2 x 10^6^ cells /ml). This time frame was chosen subsequent to the hourly late logarithmic time course. It is unclear whether the overlap in gametic/vegetative morphologies in late logarithmic cultures is due to morphological changes associated with growth, or depletion of nutrients leading to premature gametogenic induction. To minimise this risk, early logarithmic cultures were used for DNA quantification. Samples were aliquoted and centrifuged at 3000g for 3min, media was replaced with ddH_2_0. Aliquots were then pooled back into the two samples. 100ul Samples were taken each hour for 25hours and fixed in 10ul Lugol’s solution. Concentrations were measured using a haemocytometer. Every hour 1000μl of the gametogenesis replicates were centrifuged at 3000g for 3min and replaces with 70% ethyl alcohol and stored at 4°C. The day before flow cytometric analysis, cells were sedimented by centrifugation at 3000g for 5min, and supernatant replaced with 10ug/ml RNase in PBS and incubated at 37°C for 1hr. PI was added at a concentration of 15ug/ml, and maintained in the dark overnight at 4°C. Samples were filtered using 40um filters directly before analysis. For DNA analyses, 10,000 PI positive data points were collected based on singlet discrimination using PI height vs area plots. Autofluorescence profiles in the FL3 (670LPnm) channel meant that PI emission was read in the FL2 (585/42nm) channel [39]. Peaks were manually gated, and the same gates were employed on all cells. Cell concentrations did not exceed 1000cells/sec.

### Statistical analysis

Statistical analysis was performed using *IBM SPSS* Version 23 and R version 3.0.1. All flow cytometric data was tested using *FlowJo* X 10.0.7r2 (*TreeStar*, USA). Median Fluorescence and scatter trends were plotted using the ggplot2 function in R [49] with stat_smooth. Effects of nitrogen removal with respect to light phase were plotted using qplot and geom_smooth functions.

## Results and Discussion

### *Chlamydomonas* displays shifts in fluorescence and scatter properties over gametogenesis in late logarithmic populations consistent with previous investigations

**Fig 1:**
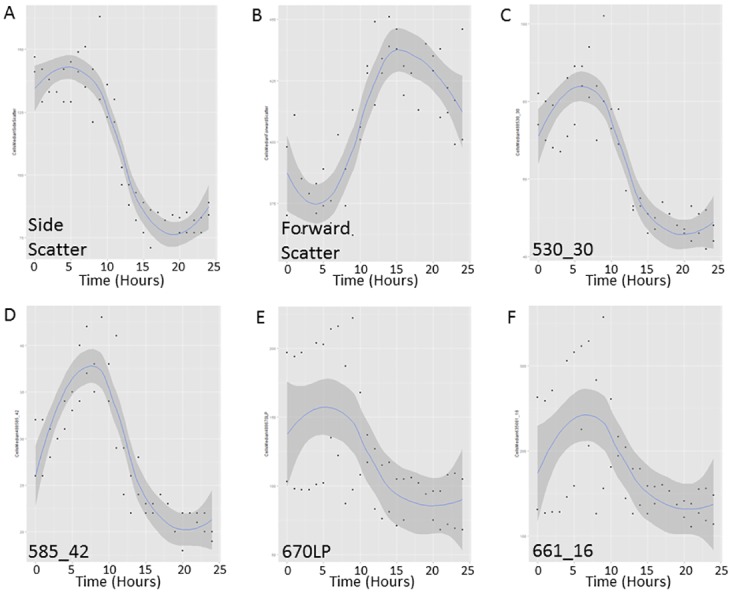
Changes in fluorescence and scatter properties in late logarithmic gametogenesis cultures. Cultures weresampled hourly over 24 hours, displayed as median values from each detector in the FACS*Calibur*. Shaded areas indicate 95% CI A) Side-scatter B) Forward-scatter C) FL1 (530_30nm) D) FL2(585_42nm) E) FL3 (670LPnm) F) FL4 (661_16nm).

**Fig 2:**
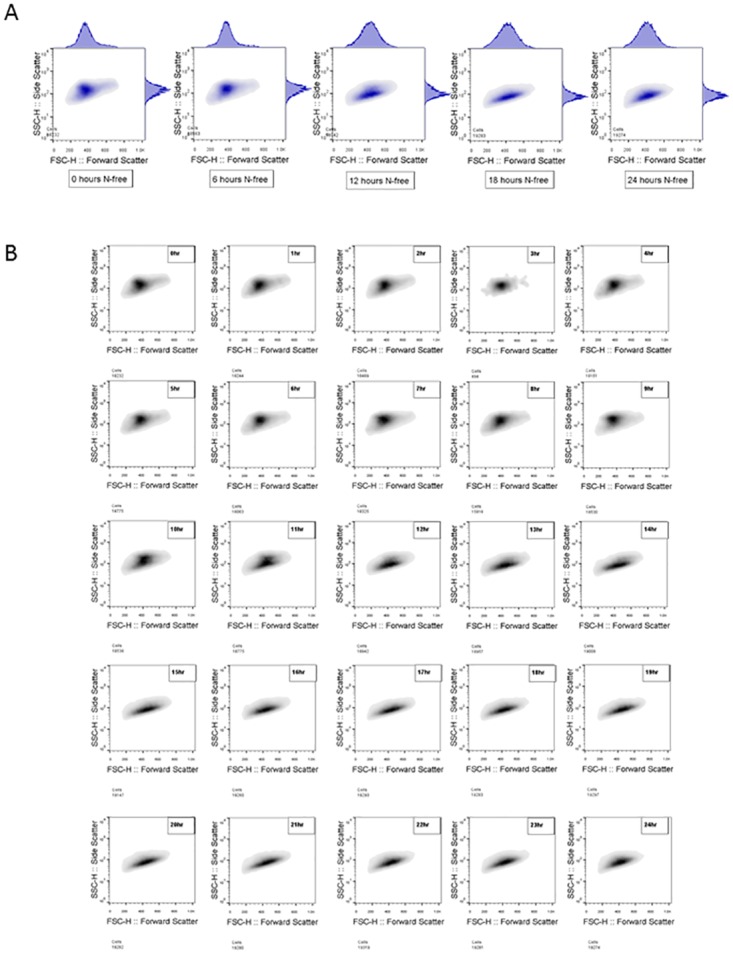
Flow cytometric measures change through gametogenesis. A) Dot plots of forward (FSC) and side-scatter (SSC) estimates, sampled every six hours over the course of gametogenesis in late logarithmic cultures. One replicate is shown B) Dot plot of forward and side-scatter, sampled hourly over the course of gametogenesis. One replicate is shown.

The time-course of gametogenesis display noticeable and reproducible changes in the scatter and fluorescence properties of the population (Fig 1), with changes occurring in specific subsets of the population over time (Fig 2).

*Chlamydomonas* gametes are smaller than vegetative cells (S4 Fig) [9], and are therefore expected to create lower levels of scatter and to contain reduced relative and absolute levels of auto fluorescent compounds. Consistent with this, side-scatter and fluorescence intensities decreased to below initial values over the final hours of gametogenesis (Fig 1), suggesting that the cells at the end of the time course are smaller and less internally complex than the vegetative cells present at the beginning of the time-course. A paired samples t-test showed that the difference in the median values of all fluorescence and scatter profiles at the removal of nitrogen and again at the end of gametogenesis, was significant only for the channel FL1 (t_1_ = 16.0 P = 0.040) and approached significance for side-scatter (t_1_ = 9.9 P = 0.064) in this small late logarithmic dataset. (df = 1 as there were two independent cultures that each had ~20,000 independent replicates, the ~20,000 replicates were combined to give a mean value for the specific timepoints tested). Testing for an increase in scatter and fluorescence over the first five hours of the trial with a linear regression, showed that there was a significant linear increase in FL2/585 channel (yellow fluorescence) (F_1,9_ = 6.577, P = 0.03) indicating that in this part of the spectrum the flow cytometer detected increases in cell complexity or other fluorescence profiles prior to cell division in this late logarithmic culture. There were no significant changes in other channels (all P values > 0.05).

Logarithmically scaled side-scatter intensities (Fig 2B) taken at hourly intervals show an overall decrease in side-scatter associated with gametogenesis, indicating a relative decrease in internal complexity, with a concentration of cells displaying high side-scatter, emerging 6 hours after nitrogen removal, and peaking around 9–10 hours. This is associated with the development at 10 hrs after nitrogen removal, of a concentration of low side-scatter cells, which increases as the high side-scatter population size decreases. This is demonstrated in both replicates and corroborates previous evidence in the literature based upon microscopic approaches in late logarithmic cultures, which showed that beyond 9 hours light after the induction of gametogenesis, genetic division occurs (doubling of genetic material before division) [35]. This would be expected to increase internal complexity (and therefore side-scatter), which in turn would decrease when the cells subsequently divide. Gametic offspring are described forming within the cell walls of adult cells around 12hrs, and by 15hours of nitrogen deprivation, are released from the parental cell wall into the media [35]. Thus the lowering of side-scatter after this time (Fig 1) is what we expect to observe. Daughter cells remain within the mother cell wall for hours post division, with the cell wall swelling, this can be determined by increases in cell volume [24]. This time frame matches our observations in the side-scatter channel (Figs 2 and 1B), with the loss of a high side-scatter population, and the drop in median scatter by 15 hour post-nitrogen removal. This may indicate that internal complexity, as measured by side-scatter, tracks cells as they increase in size prior to division.

While FL1 (530/30) and FL2 (585/42) fluorescence channels represent unknown components of *Chlamydomonas* biology, emissions detected in FL3 and FL4 correspond to chlorophyll fluorescence, emitting at 650–750 nm [50]. Changes to the number of chloroplast nucleoids are expected to change long wavelength fluorescence intensities. Examination through the process of gametogenesis, shows no increase in high FL3 or FL4 subsets (supported in Fig 1E and 1F), suggesting there is no *de novo* chloroplast nucleoid production through gametogenesis, a hypothesis which has previously been supported [51, 52]. The data corresponds to evidence that vegetative cells possess multiple chloroplasts while gametes possess a single chloroplast [52]. Therefore, the fluorescence profiles collected by flow cytometry may be able to track the production of gametes with their single chloroplast through autofluorescence intensities.

Fluorescence and scatter properties may convey meaningful information regarding the process of gametogenesis, providing a high throughput and less time-consuming method of assaying the process. However, this method is not able to detect the production of mating capable gametes, only the morphology of gametic populations. In assays of mating capacity during gametogenesis, Kates and Jones [35] documented two peaks of mating capable gametes, around 12hours (~35%) and again between 21 and 24 hours (from ~0%- to~100%). This is expected to represent the production of gametes after division of larger cells which lose mating capacity as they prepare to divide again. These dynamics cannot be determined using the fluorescence or scatter profiles described here. However, due to the morphological changes that occur during gametogenesis, scatter and fluorescence changes associated with gametogenesis may allow indirect monitoring or may complement other methods capable of estimating mating efficiency.

**Fig 3:**
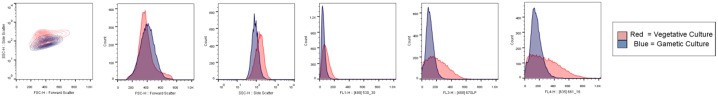
Fluorescence and scatter shifts between two differentiated cell types. Histogram display of overlap in fluorescence intensity of vegetative (immediately after re-suspension in nitrogen free media) and gametic (24hours in nitrogen free media) in late logarithmic cultures. Vegetative distributions are represented in light grey, gametic distributions in dark grey. Note overlap in all scatter and fluorescence channels.

Additionally, it is clear that while scatter and autofluorescence trends can be observed in late logarithmic cultures and used to track gametogenesis (Fig 1), overlap in the emission profiles of vegetative and gametic cell populations means that this method is less able to distinguish phenotypes for enriching gamete phenotypes in late logarithmic cultures (Fig 3). As such, we sought to explore the role that growth phase has on the ability for scatter and fluorescence analyses to distinguish vegetative and gametic cells.

**Fig 4:**
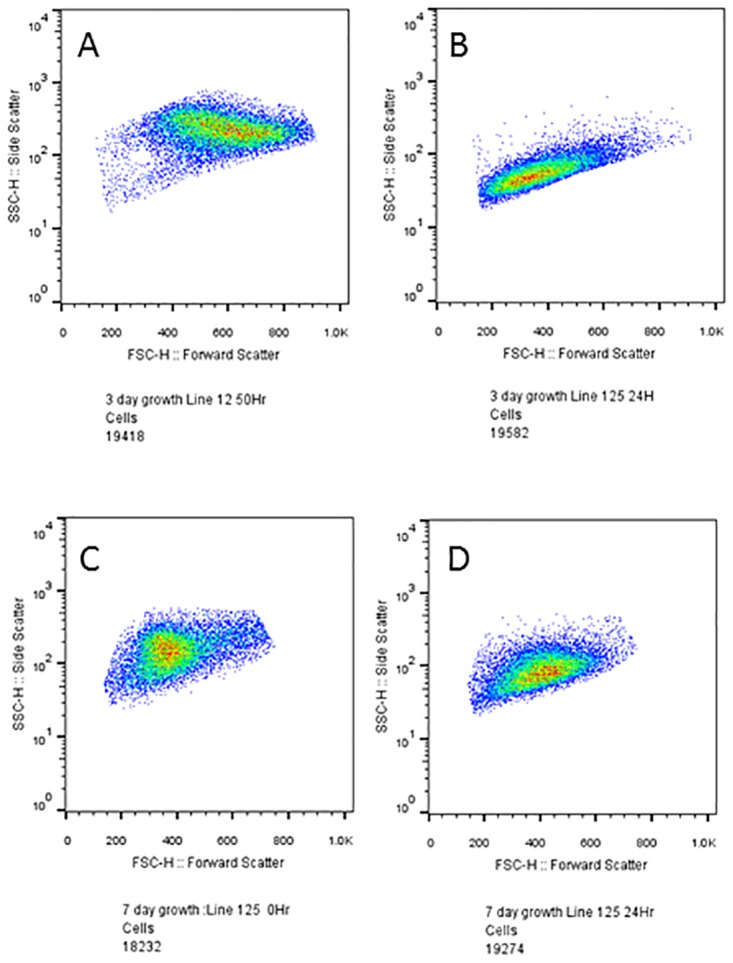
Phase of growth affects differences and overlap in vegetative and gametic morphological and fluorescence profiles. A) Early logarithmic culture immediately after transfer to nitrogen free media (i.e. vegetative cells). B) Early logarithmic culture 24hrs after transfer to nitrogen free media (i.e. gametic cells). Overlap compared to A is noticeable in forward-scatter measures (FSC), however, there is a difference in side-scatter measures (SSC) between the cell types, creating separate regions for the cell types. C), Late logarithmic culture immediately after transfer to nitrogen free media (vegetative cells) D). Late logarithmic culture 24hrs after transfer to nitrogen free media (gametic cells). Overlap compared to C is noticeable in both forward-scatter measures (FSC) and side-scatter measures (SSC) between the cell types, limiting the capacity to distinguish between cell types.

Existing literature demonstrated the association between forward scatter and cell size [53–55]. When we compare late logarithmic cultures to early logarithmic culture time courses (Fig 4) we see that the positive trend in forward-scatter of late logarithmic cultures is reversed in early logarithmic cultures, and that the difference in scatter properties between vegetative and gametic cells is increase in early logarithmic cultures. This suggests that the non-significant decrease seen in forward-scatter in the two late logarithmic time-courses (t_1_ = -4.647 P = 0.135) may be effects due to growth phase. This explains the increase in FSC (Size), when gametes should be smaller than vegetative cells. Whether this is due to cellular changes, or changes to the fragility of flagella, is unclear. In contrast, in the early logarithmic cultures, there is a significant increase in forward-scatter over gametogenesis as seen in a paired samples t-test between nitrogen removal and the end of the timecourse (t_1_ = 25.444 P = 0.025). This suggests forward-scatter can be eliminated as criteria for monitoring gametogenesis in late logarithmic cultures but that it may be of utility in early logarithmic cultures.

### Effect of growth phase on distinguishing vegetative and gametic profiles

Previous investigations suggest that the growth phase of cultures affect the number of daughter cells that can be created in multiple fission cell division, with cells dividing into 2 cells or no division at all at the end of the growth curve, compared to 2, 4, and 8 daughter cells in earlier stages of growth. Cell count data confirmed reproducible changes in the number of gamete cells produced per vegetative cell as growth progressed (Fig 4). A Generalized linear mixed model where the *vegetative and gametic cell concentrations* were the dependent variable fitted the effects of *time (day of growth)* using Poisson probability distribution and a log-link function. This model showed a significant effect of *day* (df = 7, P<0.001). Early and late in the growth curve, the ratio of vegetative to gametic cells is close to 1:1, however, in mid-growth it is between 4 and 8; as we would expect.

**Fig 5:**
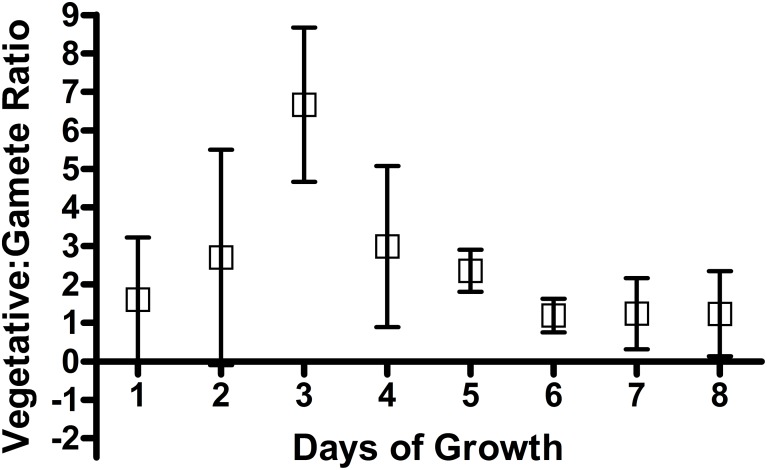
Vegetative to gametic ratio. The ratio is based on haemocytometer counts with 95% confidence intervals shown.

**Fig 6:**
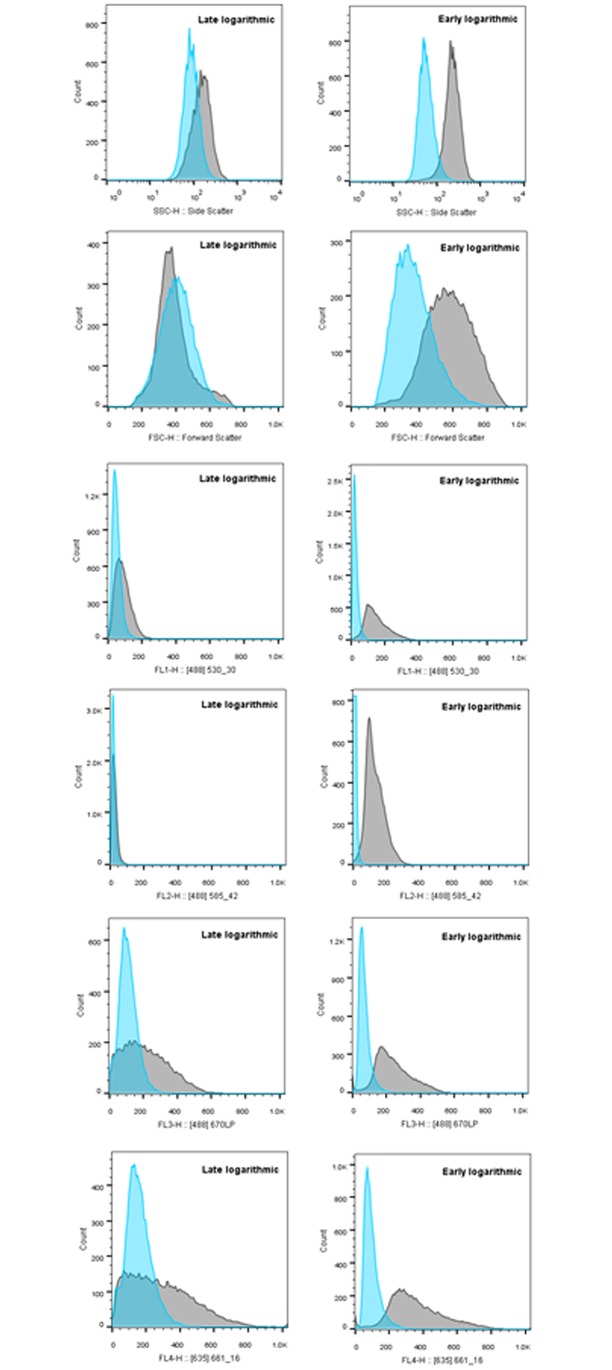
Overlay of vegetative and gametic distribution in early and late logarithmic growths. Dark Grey = vegetative cultures. Light Grey = gametic. Lower levels of overlap are seen in early logarithmic cultures compared to late logarithmic cultures. Side-scatter (SSC), FL1 and FL2 in particular show separation of the two populations.

Changes occurring over the growth phase are also reported to impact the ability of gametes to mate, with early growth phase cells not mating well [21, 35] those at the stationary phase often failing to produce quantitative (100%) mating, and cells at the end of the linear growth phase able to mate with 100% efficiency [35]. Given that growth phase affects cell division capacity, and is associated with changing nutrient availability and waste accumulation, we sought to investigate whether phases in growth differ in regard to changes in fluorescence and scatter over gametogenesis and the ability to distinguish vegetative and gametic populations based on scatter and autofluorescence profiles (Fig 5). In late logarithmic growths, there are no fluorescence channels showing complete separation between vegetative and gametic cells (Fig 6).

Side-scatter/forward-scatter plots shown in Fig 6 show the increased overlap between vegetative and gametic profiles in late logarithmic cultures compared to early logarithmic cultures. Early logarithmic growths however, show better distinction between vegetative and gametic cells based on side-scatter and other fluorescences (Fig 6). These changes are significant in many of the fluorescence channels. A paired samples t-test was used to test the difference in the median values of scatter and fluorescence profiles at the initiation and conclusion of gametogenesis in early logarithmic cultures. This analysis shows that five of the six factors showed significant changes over gametogenesis; FSC t_1_ = 25.4 P = 0.025, SSC t_1_ = 165.8 P = 0.004, FL1 t_1_ = 13.0 P = 0.049, FL3 t_1_ = 46.1 P = 0.014, FL4 t_1_ = 39.5 P = 0.016) while the FL2 channel (t_1_ = 2.1 P = 0.278) was not significant (again, df is 1 because there were two independent cultures that each had ~20,000 independent replicates, the ~20,000 replicates were combined to give a mean value at the beginning and end of the time-course for each independent culture). This suggests that further examination of the role that phase of growth can have on the morphological and fluorescent profiles in gametogenesis would be useful.

Early/mid logarithmic cultures offer a better separation of vegetative and gametic cells based on fluorescence and scatter profiles, than late logarithmic cultures (Fig 6). However, if late logarithmic cultures must be used, earlier analyses show that FL1 gives the best shift in distribution over gametogenesis as the change is significant. These data also suggest that the phase in growth can alter the trend seen in gametogenesis (See FSC and 585, Fig 6), and lower the distinction between the cell types in all channels based on morphological and fluorescence properties.

### Effect of growth phase on scatter properties of Chlamydomonas cells

Above, we have described both the general changes in scatter and fluorescence comparing both gametic and vegetative cells, as well as the effect of growth phase (Figs 4 and 6). While gamete cells tend to show lower forward and side scatter intensities than vegetative cells (due to their smaller size and lower complexity), these trends may be affected by changes in scatter associated with the growth phase (Fig 4), i.e. the population density and history of the culture, accumulation of waste products as well as progressive utilisation of nutrients.

**Fig 7:**
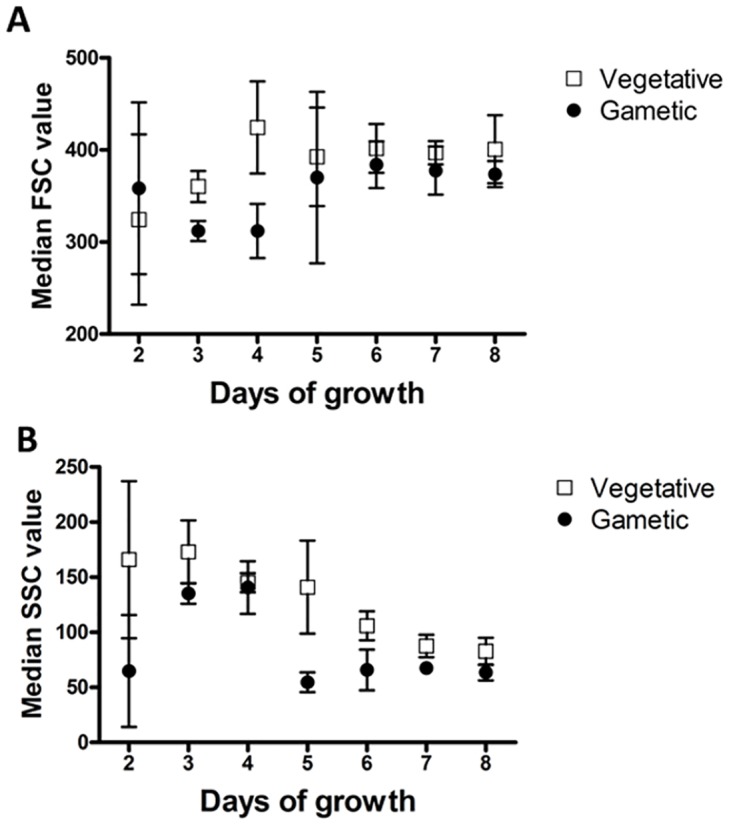
Mean FSC and SSC in vegetative and gametic profiles across the growth phase.

To investigate the role of growth phase, we sampled vegetative and gametic *Chlamydomonas* cells across the growth curve (Fig 7). This replicated previous findings that late logarithmic cultures show increased overlap in scatter profiles of vegetative and gametic cells at later phases of growth, and show variation in the number of gametes produced (S5 Fig). Interestingly, we found that forward scatter values showed overlap in early growth cultures, which separated at early/mid logarithmic concentrations, and overlapped again towards higher concentrations. This showed that vegetative size increased to a consistent level from 4 days growth, whereas gametic cells showed lower sizes in early phases, and increased to a consistent size from day 5.

In contrast, the side scatter profiles of both gametic and vegetative cells decrease over the growth phase. There is a point at which the side scatter of gamete cells decreases to a constant level in the mid and later phases of growth. We have previously seen that for every vegetative cell, the number of gametes produced changes depending on the phase of growth (Fig 5). This may explain the difference in scatter between vegetative and gametic cells, with cells at early and late phases of growth showing direct differentiation from vegetative to gametic cells, without division leading to larger gametes on average. These results also raise questions as to the reasons behind the overlap in scatter of the cell types at later phases of growth and question the utility of using late logarithmic cultures specifically for assessing the morphological differentiation of gametes (i.e. measuring the propensity for mating). The question remains, are these similarities between early and late logarithmic scatter profiles due to cell growth forces that lead to differentiation without division, or are late logarithmic cultures already differentiated into gametes due to depletion of nutrients associated with cell growth in confined media?

### DNA replication over gametogenesis

**Fig 8:**
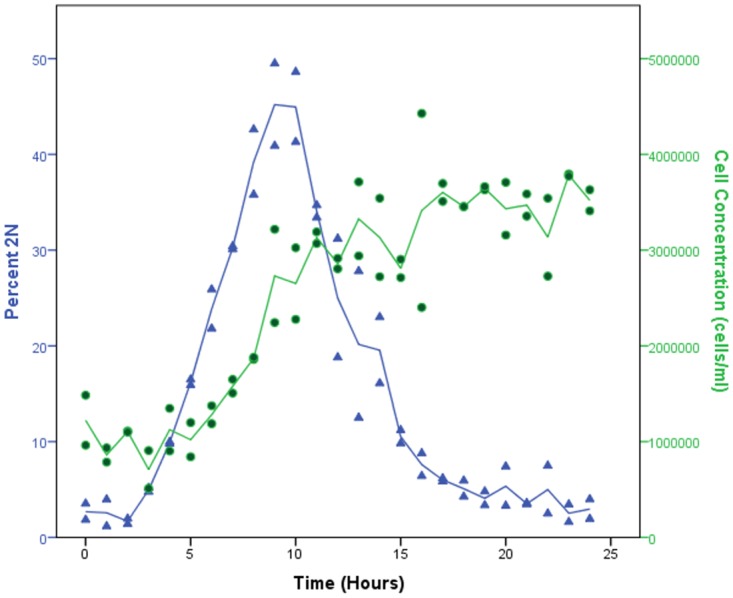
Cell division in early logarithmic cultures. Comparison of diploidy percentage with haemocytometer counts of gametogenesis ploidy time course. The largest increase in the cell population occurs prior to the peak in diploid cells, slowing as cell division ceases. Fitted with straight interpolation line in SPSS.

Previous investigations into the process of cell division during the production of gametes, describes the morphological identification of DNA multiplication [35]. In order to create a sensitive quantification of this process in *Chlamydomonas*, we employed Propidium Iodide (PI) staining, since PI fluoresces when intercalated in DNA [56]. Microscopic analyses suggest that after 9 hours in light after the induction of gametogenesis, genetic division occurs (doubling of genetic material before division) [35]. In our flow cytometric time course we see diploid levels peaking at 9 hours, but the beginning of DNA synthesis starting as early as 4 hours after the removal of nitrogen with rates of diploidy beginning to drop at 11hrs- 14hrs (Fig 8). This suggests variation in the initiation of cell division between cells.

Microscopic approaches [35] confirm that gametic offspring form within the adult cells wall around 12hrs, and by 15hours of nitrogen deprivation have been released from the parental cell wall into the media. This corresponds to the end of the peak seen in side-scatter and ploidy fluorescence prior to 14–15 hours post nitrogen removal. We see a drop in diploidy around 15hours, which may confirm loss from parental cell walls, however, the distribution of diploidy continues to fall after 15hours, suggesting some cells may still be being released from parental cell walls, and that there is variation in this process.

### The relationship between nitrogen removal and light phase on the process of gametogenesis

**Fig 9:**
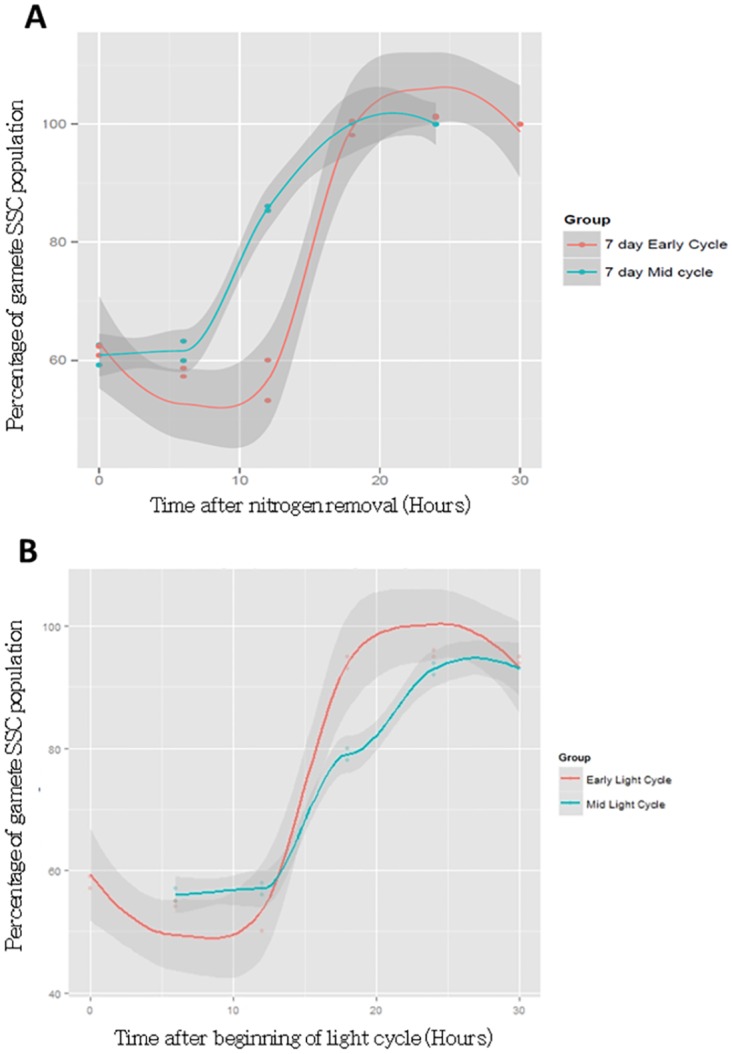
Phase in light cycle at which nitrogen is removed influences the dynamics of gametogenesis. A) Comparison of percentage of cellular events occupying the gametic gate (low Side-scatter gated on gametic population) over the course of gametogenesis, with respect to the time since nitrogen removal. B) Comparison of percentage of cellular events occupying the gametic gate over the course of gametogenesis, with respect to the light cycle.

Mating reactions suggest that light phase impacts the process of gametogenesis [35], and so we measured the effect that nitrogen removal at the beginning or middle of the light phase had on the percentage of cells occupying the gametic side-scatter distribution in the time-course. We used a generalized linear mixed model to test the effect of the time in the light cycle that nitrogen was removed, on the proportion of cells in the ‘gamete’ side-scatter gate over the course of gametogenesis. We specified the treatments ‘*time into the light-cycle*’ (that nitrogen was removed), and ‘*time since*’ (the removal of nitrogen) as fixed factors, *replicate* was a random factor and was nested within *‘time into the light-cycle’*. The whole model was significant (F_10,10_ = 4,721, P<0.001), there was a significant *time into the light-cycle* by *time since* interaction (F_4,10_, = 865.9 P<0.001) and so the main effects of *time into the light-cycle* (F_1,10_ = 7.39, P = 0.02) and *time since* nitrogen (F_4,10_ = 866.0 P<0.001) are not interpreted further. The variance due to *replicate*(*time into the light-cycle*) was not significant (Z = 0.95, P = 0.341). Comparing the process of gametogenesis from the time nitrogen is removed (Fig 9A), cells induced in the middle of the light cycle show a faster production of gametes than those induced at the beginning of the light cycle. Whether this creates an earlier plateau in gamete production is not clear given the resolution of the time course. The altered slope of the distribution (Fig 9) is interpreted as the requirement to reach a certain size to produce gametes, and as cells are smallest near the beginning of the light cycle, they require a period of growth before gametogenesis. By comparing percentage gamete formation to light phase (Fig 9B), gamete formation appears to begin at the same point in the light cycle (around 12–13hours after light initiation), but cells induced in the middle of the light cycle form gametes at a higher rate.

### Effective Scatter and fluorescent properties for identifying gamete sub-populations

Evidence from three experiments; the late logarithmic time course, early logarithmic time course and early logarithmic replication experiment, confirm that the scatter and fluorescence channels differ in the extent to which vegetative and gametic cells overlap in their profiles. In early logarithmic cultures there is also evidence of shifts in the distribution of fluorescence with low levels of overlap especially in side-scatter and to a lower extent FL1(530/30) and FL2 (585/42) (Fig 6). This corresponds to the t-tests reported earlier, in which significant changes in early logarithmic cultures were found in all channels except FL2. Chi squared tests on raw cell counts in uniform SSC and FL1 gates (based on gamete cell profiles) collating all three replicates in early logarithmic cultures were performed to determine if the difference between post transfer and gametic cultures were significant in early logarithmic cultures. Changes in side-scatter χ^2^_1_ = 41295, and FL1 χ^2^_1_ = 42603.06, showed highly significant changes (P > > 0.05).

## Conclusion

**Fig 10:**
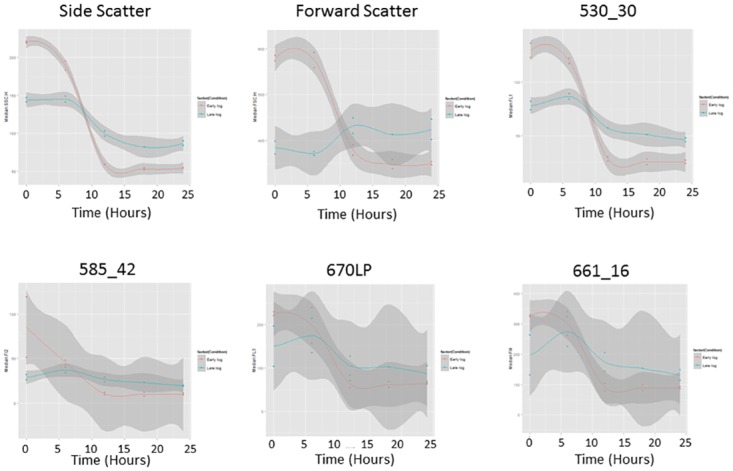
Smoothed plots comparing median fluorescence and scatter properties over gametogenesis in early and late logarithmic cultures. Samples taken every six hours are displayed. Shaded areas indicate 95% CI

In summary, we report a novel and high-throughput method for indirectly monitoring population variation in the process of gametogenesis in *Chlamydomonas reinhardtii*; a method compatible with existing approaches to studying gametogenesis and the acquisition of mating competence. Flow cytometric time courses revealed significant changes in scatter and fluorescent profiles, associated with gametogenesis, narrowing down scatter and fluorescence criteria for enriching gamete phenotypes (SSC/FL1 in early logarithmic cultures and FL1 in late logarithmic cultures). This high throughput approach allowed investigation of the dynamics of gametogenesis, such as the impact of light phase and therefore cell size and growth on the rate at which cells displaying a gametic phenotype emerge. Changes documented throughout gametogenesis were able to provide scatter and fluorescent profiles for future cell sorting or comparisons of gametogenesis, and display the utility of autofluorescence in tracking cell changes. Finally, we have demonstrated the complex relationship between growth phase (Fig 10), cell morphology and differentiation; raising questions about the validity of using late logarithmic cultures in assessments of cell specification.

The overlap between vegetative and gametic distributions, in addition to the similarity between early logarithmic gametes and late logarithmic vegetative cells (Fig 4), raises further important questions. It is unclear whether the lowered median side-scatter of late logarithmic vegetative cells are due to cell competition, nutrient limitation, waste accumulation or other factors. For example, we can expect that as cells approach stationary phase, nutrients will decrease in abundance and waste products will accumulate. Given that nitrogen can be depleted in plate cultures over a few days [9], a similar process may occur in liquid cultures, therefore the hypothesis that late logarithmic cultures may include a subset of already differentiated gametes, does require investigation. In the related species, *Chlamydomonas eugametos*, where gametogenesis is not cued by nitrogen, but another nutrient stressor, mating capable gametes have been observed to develop later in the growth phase as nutrients become limiting [46]. If this is true for *Chlamydomonas reinhardtii*, mating efficiency tests using late logarithmic cultures (a common experimental strategy) would not give a repeatable measure of mating efficiency. Further complications of the process of gametogenesis in late logarithmic cultures come from the observations of [35] who noted that above 3 x 10^6^ cells /ml, cells can lose synchronisation. Non-synchronous cultures show a different distribution in the rate of gamete production, which might also contribute to the quantitative mating capacity seen in late logarithmic cultures [35]. This could be further investigated by testing the mating capacity of vegetative cultures at different phases of growth.

## Supporting Information

S1 Fig Effect of centrifugation on scatter and fluorescence. (TIF)

S2 Fig Live;dead staining (Propidium Iodide) in *Chlamydomonas reinhrdtii* strain CC125. (TIF)

S3 Fig Proof of method for detecting ploidy utilising synchronisation of cell division. (TIF)

S4 Fig Sizes of gametic and vegetative cells based on relative size collected by *CASA* data. (TIF)

S5 Fig Mean Cell concentrations of vegetative, centrifuged and gametic cells (each with 3 replicates) across the growth phase. These replicates were used for flow cytometry, comparing scatter profiles across the growth phase.
(TIF)
