# Supplementary figures and images for: Flow Cytometric Methods for Indirect Analysis and Quantification of Gametogenesis in Chlamydomonas reinhardtii (Chlorophyceae)
Source: PLoS One. 2016 Sep 27;11(9):e0161453. doi: 10.1371/journal.pone.0161453 (PMC5038954; doi:10.1371/journal.pone.0161453)

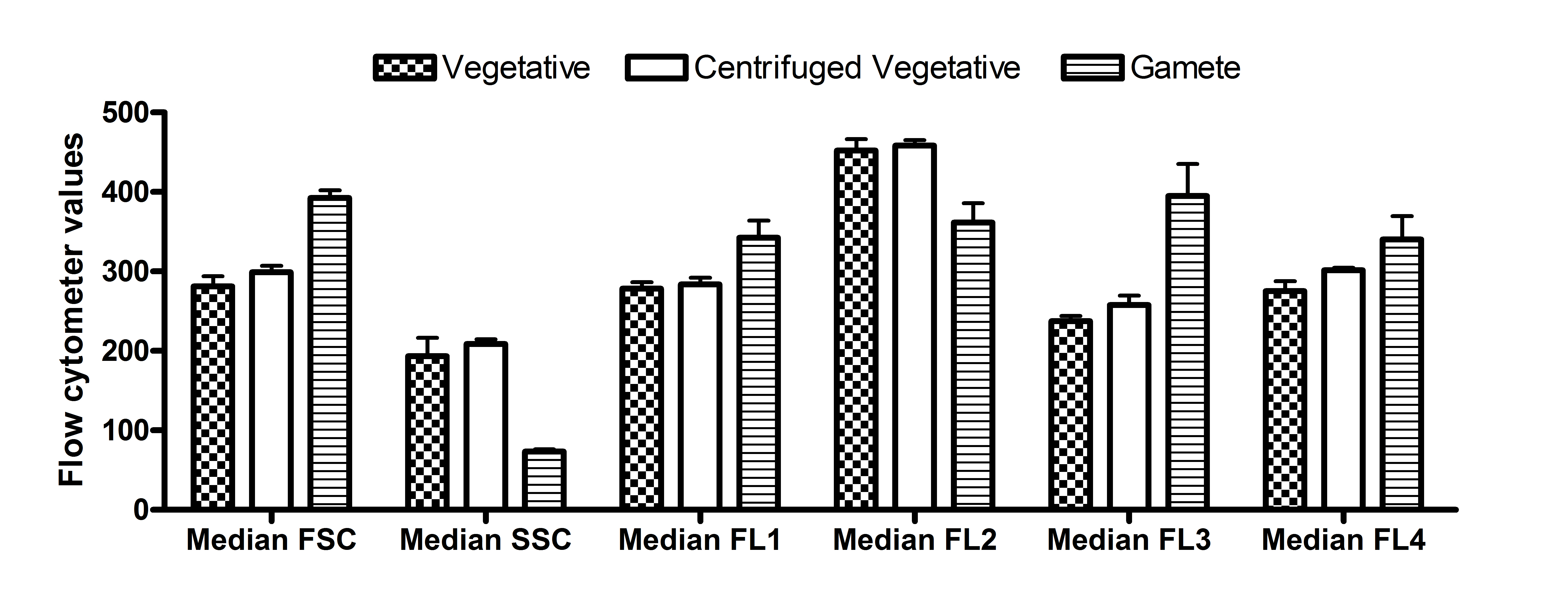

Supplement: S1 Fig — (TIF) [file pone.0161453.s001.tif]

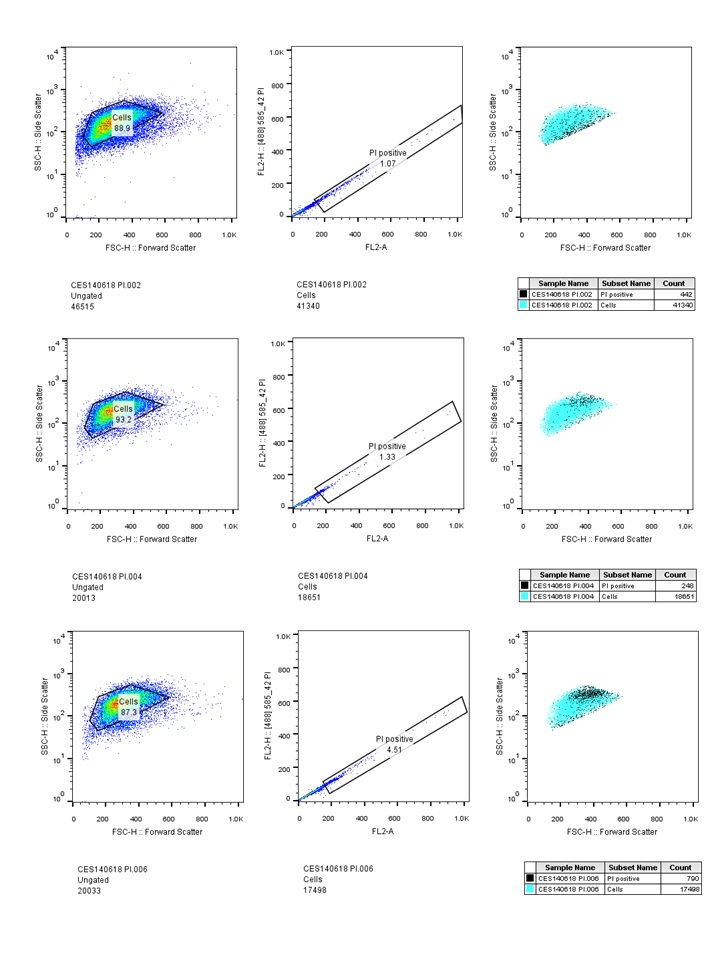

Supplement: S2 Fig — (TIF) [file pone.0161453.s002.tif]

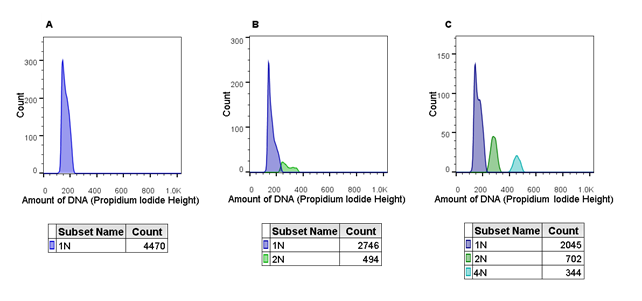

Supplement: S3 Fig — (TIF) [file pone.0161453.s003.tif]

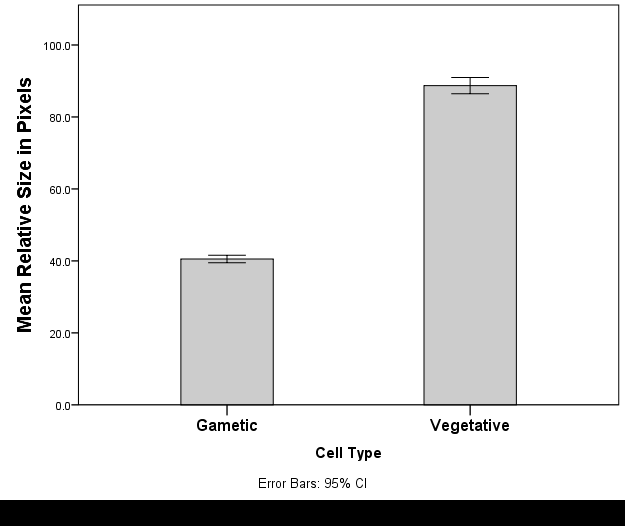

Supplement: S4 Fig — (TIF) [file pone.0161453.s004.tif]

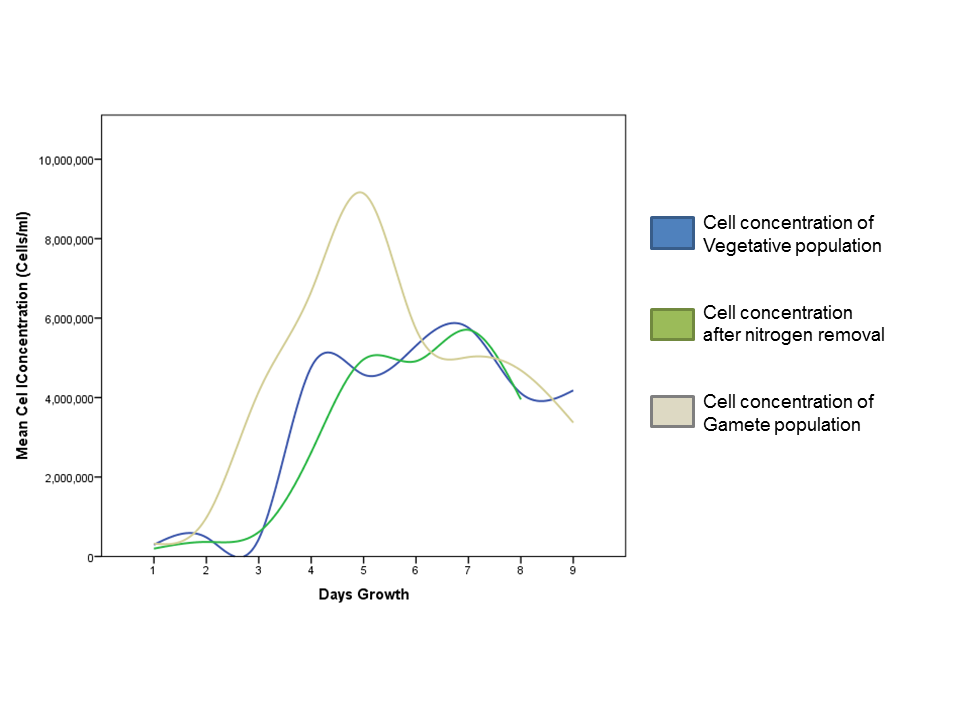

Supplement: S5 Fig — These replicates were used for flow cytometry, comparing scatter profiles across the growth phase. (TIF) [file pone.0161453.s005.tif]
